# Chopped or Long Roughage: What Do Calves Prefer? Using Cross Point Analysis of Double Demand Functions

**DOI:** 10.1371/journal.pone.0088778

**Published:** 2014-02-18

**Authors:** Laura E. Webb, Margit Bak Jensen, Bas Engel, Cornelis G. van Reenen, Walter J. J. Gerrits, Imke J. M. de Boer, Eddie A. M. Bokkers

**Affiliations:** 1 Animal Production Systems Group, Wageningen University, Wageningen, Netherlands; 2 Department of Animal Sciences, Aarhus University, Tjele, Denmark; 3 Biometris, Wageningen University, Wageningen, Netherlands,; 4 Livestock Research, Wageningen University and Research Centre, Lelystad, Netherlands; 5 Animal Nutrition Group, Wageningen University, Wageningen, Netherlands; Utrecht University, Netherlands

## Abstract

The present study aimed to quantify calves'(*Bos taurus*) preference for long versus chopped hay and straw, and hay versus straw, using cross point analysis of double demand functions, in a context where energy intake was not a limiting factor. Nine calves, fed milk replacer and concentrate, were trained to work for roughage rewards from two simultaneously available panels. The cost (number of muzzle presses) required on the panels varied in each session (left panel/right panel): 7/35, 14/28, 21/21, 28/14, 35/7. Demand functions were estimated from the proportion of rewards achieved on one panel relative to the total number of rewards achieved in one session. Cross points (cp) were calculated as the cost at which an equal number of rewards was achieved from both panels. The deviation of the cp from the midpoint (here 21) indicates the strength of the preference. Calves showed a preference for long versus chopped hay (cp  = 14.5; *P*  = 0.004), and for hay versus straw (cp  = 38.9; *P* = 0.004), both of which improve rumen function. Long hay may stimulate chewing more than chopped hay, and the preference for hay versus straw could be related to hedonic characteristics. No preference was found for chopped versus long straw (cp  = 20.8; *P* = 0.910). These results could be used to improve the welfare of calves in production systems; for example, in systems where calves are fed hay along with high energy concentrate, providing long hay instead of chopped could promote roughage intake, rumen development, and rumination.

## Introduction

Foraging animals gather information about available resources at the expense of optimising immediate rate of energy gain [1], [2]. Ruminants have been found to trade-off between optimising rate of energy gain and minimising disadvantages to rumen function caused by the intake of high energy food, by including in their diets roughage high in fibre and low in energy [3], [4]. This requires prior association between the sensory characteristics of feed and their post-ingestive consequences [5]. Ruminants spend extensive time feeding and ruminating. Mastication and rumination promote salivation, an important buffering agent in the rumen, and reduce feed particle size to enable passage of feed into the abomasum [6], [7]. As a consequence, ruminants have a high incentive to chew and ruminate [8], [9], and they may sometimes show a preference for roughages that require long chewing times [10]. The latter is especially relevant in farmed ruminants fed high energy diets with little fibre, as these animals develop abnormal oral behaviours due to limited opportunity to chew and ruminate [11]–[13]. Abnormal behaviours occur in sub-optimal environments and are a sign of poor welfare in captive animals [14].

A method for investigating foraging behaviour in ruminants is to quantify the preferences for two simultaneously available feeds. Manipulating the particle length of roughage is an easy way to control the rate of energy gain, without affecting taste and smell. Compared to longer ones, smaller particles of roughage are ingested at a higher rate [15]–[19], and pass faster/more easily through the reticulorumen [20], resulting in an increased rate of energy gain. However, feeding only small amounts of small particles of roughage, as opposed to longer roughage particles, on top of a high concentrate diet, may lower ruminal pH in the long term, increasing the chances of developing acidosis [7]. These diets may also lead to ruminal plaque formation, i.e. a sticky mass of hairs and small feed particles between the papillae [21], and ruminal hairball development [13]. In addition, small roughage particles often mean less chewing and rumination than longer particles. Less chewing and rumination increases energy intake rate by decreasing ingestion and digestion effort, but these behaviours also stimulate saliva secretion, which is an important buffering agent in the rumen [7]. Ruminants were capable of making foraging choices that favour good rumen function by selecting a large portion of chopped roughage particles (30%) in their total diet, when chopped and ground roughages were offered together [3], [4]. In previous studies, however, animals had to balance energy intake and good rumen function, because no other feed was provided besides roughage. If energy intake was taken out of the equation, by, for example, feeding high energy concentrate, ruminants are expected to prefer longer particles of roughage, as the need for good rumen function would then become more important than rate of energy gain.

Previous research investigating preferences for different particle lengths of roughage in ruminants used short-term [18], [22] or long-term [3], [4] choice tests. Providing freely available alternative resources and imposing no cost on preference, however, does not reflect foraging environments in the wild and does not quantify the strength of a given preference. Cross point analysis of demand functions, where two substitutable resources are presented simultaneously and the workload for each resource is varied relative to the other, incorporates a ‘cost’ on the choice and is suggested as a more accurate and biologically relevant method for quantifying preferences [23], [24]. In this method, demand function refers to the linear regression between rewards achieved and resource costs [25]. The cross point designates the combination of costs (one for each resource) at which an equal number of rewards is achieved for both resources. The cross point analysis of double demand functions enables quantification of preferences, and may be viewed as reflecting the natural foraging situation where food availability (cost) varies.

The present study aimed to quantify calves'preference for long versus chopped hay and straw, using double demand operant conditioning, in a context where energy intake was no limiting factor (i.e. feeding large quantities of milk replacer and concentrate). We hypothesised that calves would prefer long roughage particles over chopped because they value long chewing time and good rumen function. This presupposes that calves previously learnt post-ingestive consequences of different roughage types. Hay is associated with increased energy intake rate and better rumen function [26], but decreased chewing time [22], compared to straw. Moreover, sensory characteristics, such as smell, taste or texture, may also affect the relative preference of hay and straw. The preference for hay and straw was also quantified in the present study.

## Materials and Methods

This study was carried out at Wageningen University's Animal Science Department experimental facilities, The Netherlands. The experiment ran from April to August 2012.

### Ethics statement

All procedures met the terms of the Dutch law for animal experiments, which complies with the ETS123 (Council of Europe 1985 and the 86/609/EEC Directive), and were approved by Wageningen University's Committee on Animal Care and Use (DEC no. 2012006).

### Animals and husbandry

**Figure 1:**
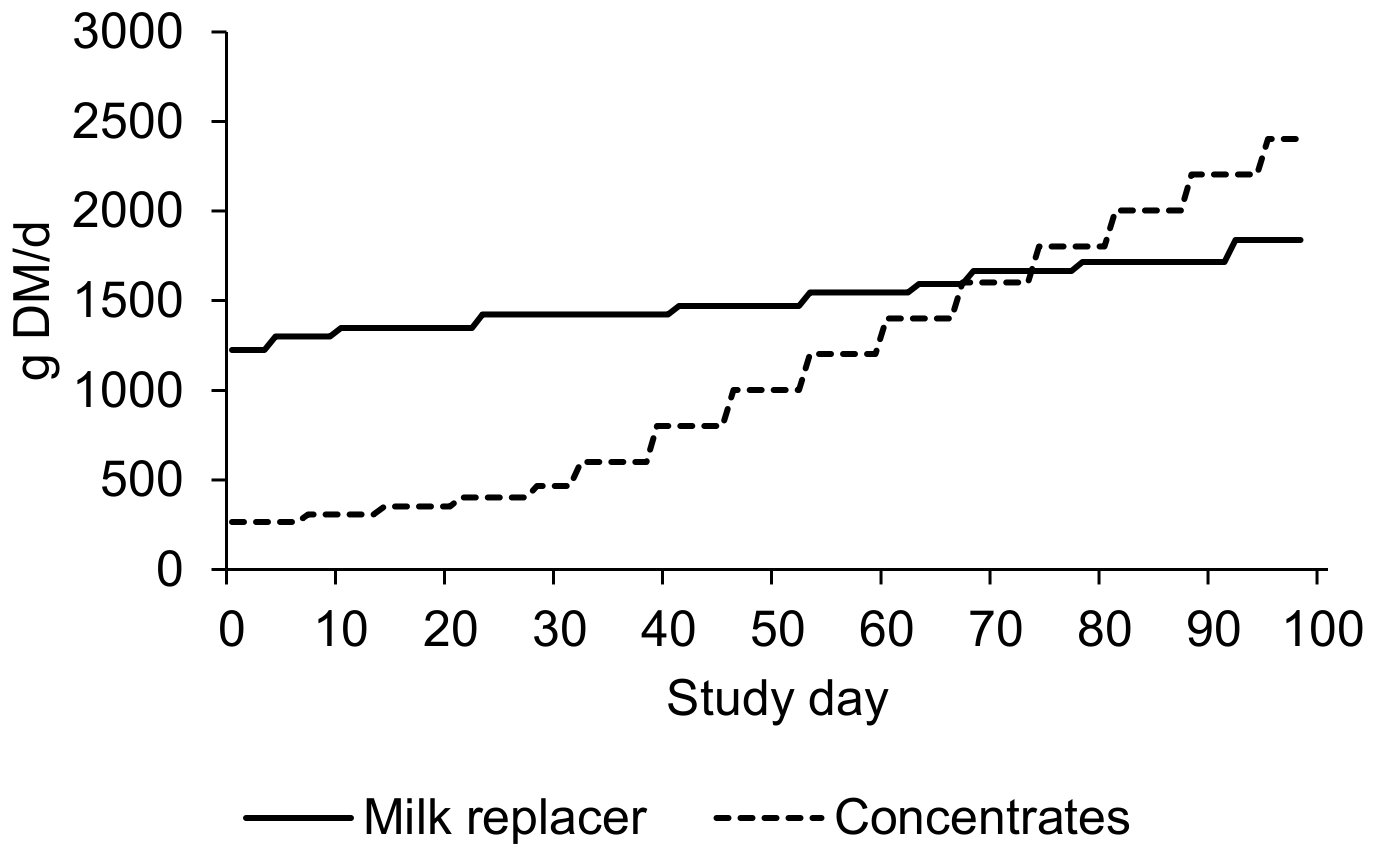
Milk replacer and concentrate feeding. Feeding schedule for milk replacer and concentrate in g DM per day per calf. Milk replacer was fed in two meals per day at 07:30 and 16:30 h, whereas concentrate were fed only at 16:30 h. Milk replacer and concentrate were fed in buckets, with floating teats for the milk.

Nine 7-week-old Holstein-Friesian bull calves (body weight mean ± SEM: 84.6±1.3 kg) were purchased from one Dutch veal farm. Calves were individually housed for the first 4 weeks after arrival at the veal farm (from 2 to 6 weeks of age), and thereafter, housed in a large group of 95 male calves. On the veal farm, calves had access to brushes (for grooming), bouncy balls (for head butting), and rubber teats (for sucking and chewing). The calves were fed milk replacer, concentrate (400 g per calf) and a small amount of chopped wheat straw (10 g per calf). The calves for the experiment were selected based on two criteria: similar size and no previous health treatment. At arrival at the experimental facilities, the nine calves were housed together in one 9.40 m×2.45 m home pen with a wooden slatted floor. The home pen was fitted with two brushes (for grooming) and one plastic ball hanging from a chain for enrichment. The calves received commercial milk replacer (18% crude protein and 18% crude fat) twice a day at 07:30 and 16:30 h in buckets with floating teats. Calves were also fed pelleted concentrate (17.5% crude protein, 37% starch, 24% NDF, based on 71% cereal and cereal by-products and 25% lupins as the main ingredients), which were provided once a day in the milk buckets immediately after the milk was consumed during the afternoon feeding. All calves finished their milk meal within 10 min. Calves were restrained during milk feeding to prevent them from ingesting other calves' milk. The daily allowance of milk replacer and concentrate corresponded to ad libitum intakes of these feeds in similar age calves in a previous study, where milk replacer, concentrate, maize silage, hay and barley straw were offered ad libitum (unpublished data). The allowance of milk replacer ranged from 10.0 L/d at 7 weeks of age to 15.6 L/d (122 g DM/L) at 5 months of age, while the allowance of concentrate ranged from 0.3 kg/d at 7 weeks of age to 2.7 kg/d at 5 months of age (Figure 1). The choice of the feeding strategy (milk fed twice a day and concentrate fed only at night) enabled control of intake before testing. After arrival, calves were offered five roughages: chopped barley straw, long barley straw, chopped grass hay, long grass hay (straw: 3.1% crude protein and 79% NDF; hay: 9.2% crude protein and 59% NDF), and chopped Lucerne hay mixed with 8% cane molasses and linseed oil (molashine, Gedizo Trading Int.). Chopped roughage particles were 2–3 cm, while long particles were unprocessed and around 20–30 cm. These particle lengths were chosen as providing the largest possible variation in length, with the smaller length reflecting what is commonly fed to fattening calves. The five roughages were offered one after the other in order to familiarise the calves with sensory and post-ingestive information associated with each roughage type. This familiarisation was done for three consecutive days per roughage type (i.e. 15 days of familiarization in total starting the day after arrival), offered ad libitum. After this initial familiarisation period, calves only received roughage (i.e. long and chopped hay and straw) in the home pen during days with no training or days with no testing. During the training period, which lasted a total of 6 weeks, calves were not brought into the operant pen during the weekend, i.e. there were 2 d/wk without training. During the testing period, which also lasted 6 weeks, the Sundays were used for habituation to the new roughage types on a low workload, i.e. there was 1 d/wk without testing (see subsection “Testing calves” below). All test-roughages (i.e. all roughage types except Lucerne hay) were offered in the home pen each weekend. Roughage intake in the home pen during familiarisation and during days without training or testing was recorded.

Milk and concentrate refusals in the home pen were weighed daily. Milk refusals only occurred once (on the day of arrival at the experimental facilities). Concentrate refusals were less than 5% of provision, on average, throughout the study. The calves received water ad libitum via two drinking nipples. Lights were on between 07:00 and 22:00 h. Temperature was regulated with a heater and mechanical ventilation, and ranged from 14.4 to 26.1°C. Relative humidity ranged from 50.6 to 97.1%. A radio was turned on during the day in an attempt to maintain constant ambient background noise. In the week after arrival, calves were blood sampled for haemoglobin (Hb) and serum iron (SeFe) analysis in order to ensure that they were not anaemic: (mean ± SEM) Hb  = 6.8±0.1 mmol/L and SeFe  = 36.3±3.2 µmol/L. Given these values, calves were not given extra iron.

In order to test the equipment and develop a training protocol for the calves in this study, a pilot study was conducted using three calves prior to the present study.

### Training calves on double demand operant conditioning

The test pen (2.35 m×2.45 m) was immediately adjacent to the home pen, fitted with a wooden slatted floor and black plastic walls (1.45 m high), and accessible from the home pen through a door. Calves could, therefore, be walked from the home pen, through the door, into the test pen. On the wall opposite the door were two panels (24 cm×20 cm) and two buckets (33 cm diameter). The two buckets were located between the two panels. Each bucket was 17 cm away from the corresponding panel, and the distance between the two buckets was 53 cm. The panels were raised 60 cm above the floor and the bottom of the buckets were raised 46 cm above the floor. Above the buckets were cylindrical automated feed delivery systems with a clap that opened to release roughage rewards into the buckets, via a computer that recorded the number of successful presses made on the panels. The left panel and bucket were associated to each other, in such a way that the correct number of presses on the left panel would result in the delivery of a roughage reward into the left bucket. The same applied to the right panel and right bucket. When panels were active, that is when the computer system was switched on, panels were lit with white led lamps. Each successful press made to an active panel was rewarded with a bell sound. When a reward was delivered, an alarm sound was played and the lights in both panels went off for 500 ms.

The nine calves were randomly assigned to groups of three, and randomly assigned to a working order within each group. During the entire experiment, including habituation, shaping, training and testing, calves were always placed in the test pen in the same order so that they could form expectations as to when they would be given the opportunity to work for roughage. One section of the home pen, adjacent to the test pen, could be closed off and formed a “waiting room” (2.35 m×2.45 m). To avoid disturbing all calves every time a new calf was collected for testing, calves were placed in the waiting room in their groups of three and remained there until all three calves had visited the test pen. Calves were first habituated to the test pen in their groups of three for 10 and 30 min. They were then habituated to the test pen individually for 10 and 20 min. Each calf visited the test pen once per day. During all habituation sessions, except the last two, the panels were inactive, meaning that the lights in the panels were off and a muzzle press resulted in neither sound nor reward. In the last two habituation sessions, the panels were active in order for calves to habituate to the lights in the panels. One muzzle press resulted in reward delivery.

During shaping and training, the reward on both panels was 10 g of Lucerne hay. During shaping, one panel and its corresponding bucket were blocked off with a barrier, and calves could only access the other panel and its corresponding bucket. During shaping, calves were rewarded for the following behaviours in the following sequence: approach the panel, sniff the panel from any angle, sniff the top of the panel, touch the top of the panel with the muzzle, and press the panel. When calves successfully learnt to press the panel to gain access to a reward, they were shaped on the other side. The side made accessible first was balanced for each group of calves.

Once calves were shaped on both panels, the fixed ratio (FR), i.e. number of presses required for one reward, was increased to two (FR2). After this, the barrier was removed and calves were trained on both panels, which were accessible simultaneously, on FR2. Subsequently, the FR on both panels was gradually increased while maintaining the same FR on both panels until FR10. Finally, the difference in FR between the two panels was gradually increased until calves could be trained on the five FR pairs used during testing: (Left-right panel) 7/35, 14/28, 21/21, 28/14, 35/7. Training ended when all calves worked economically, i.e. accessed over 60% of rewards from the panel with the lowest FR. At this stage, calves were 15 weeks old. Training sessions lasted a minimum of 30 min, but no maximum duration was imposed on the calves. This was done to enable all calves to work at their own individual speed and to access the number of rewards that they were motivated to get. Training sessions were ended when the calves had received no rewards for 3 min, after the initial 30 min. Training sessions lasted 39 min on average. For testing sessions, the minimum session time was reduced to 20 min, but again no maximum session time was imposed. When calves did not receive a reward for 3 min between 20 and 40 min in the test pen, or when calves walked away from the panels after 40 min in the test pen, the session was ended. Testing sessions lasted 39 min on average. Therefore, changing the criteria used during training did not affect average session duration.

### Testing calves

Calves' preference for three combinations of roughage types was tested, and each combination was tested for 2 weeks: 1) chopped hay versus long hay, 2) chopped straw versus long straw, 3) chopped hay versus chopped straw. Each week comprised of one day of habituation with FR7 on both panels (to allow calves to familiarise themselves with the two roughage types and the location of each type) and five testing days; i.e. one day per FR pair: (Left-right panel) 7/35, 14/28, 21/21, 28/14, 35/7 presented in a random order). The two weeks with the same combination were repetitions of each other, but the location of the two roughage types was switched in order to control for any pre-existing side bias. The first two combinations of roughage types, which both investigated preference for different particle lengths, were presented in a cross-over design, with half the calves starting with chopped versus long hay and the other half starting with chopped versus long straw. After this, calves' preference for hay versus straw (both chopped) was tested. During testing of chopped versus long roughage, the reward size was 5 g, whereas during the testing of hay versus straw, the reward size was 8 g. The reward size was increased in an attempt to reduce test session duration and to take into account the older age of the calves. If calves did not consume all rewards, refusals were weighed at the end of the session and noted for each roughage type. The number of rewards used in the analysis was based on consumed rewards (number of rewards delivered minus number of rewards not consumed).

### Post-mortem measurements

In order to check for any underlying health issues that may have affected the preferences of calves for different types of roughage, post-mortem health measurements were collected. At 6 months, all calves were slaughtered in a small slaughter house and routine Welfare Quality® post-mortem measurements were carried out [27]. Respiratory and gastrointestinal health measurements were made on all calves. Pneumonia was scored from 0 to 3 based on damaged area on the lungs, and presence of pleuritis was noted. Plaque and hyperkeratosis in the rumen, as well as lesions in the torus pylorus and pylorus areas of the abomasum were noted as present or absent. Rumen development was scored from 1: low to 4: full. A rumen score was calculated as the median of the rumen scores on the 9 rumens. Damage from abomasal lesions of <0.5 cm^2^ (category 1), 0.5–1.0 cm^2^ (category 2), and >1.0 cm^2^ (category 3), were scored from 0 (absent) to 4 based on the number present. An abomasal lesion score was calculated for each calf as the sum of the lesion number, multiplied by the lesion category. The median of these scores was then calculated.

### Data analysis

The response variable was the proportion of rewards of one resource over the total number of rewards for both resources within a session. This choice for a response variable differs from previous studies using cross point analysis of double demand functions, which generally used (logarithms of) reward counts [23], [24], [28]–[30]. We suggest that using proportions is more appropriate, as it takes into account the dependence between two simultaneously presented resources. A two-step approach was followed where (1) a model was fitted to the data of each individual animal and individual cross points were estimated, and (2) these individual cross points were compared to the midpoint. The midpoint in the present study was 21, i.e. the point where the FR values for the two resources were the same.

The two-step approach circumvented the need for modelling a dependence structure between proportions of the same animal over different sessions (resulting from repeated measures design). The model fitted to the data per animal was a generalised linear model (GLM) [31] with a logit link, the variance was specified as a multiple of the binomial variance function, and FR (of the chopped reward or of the hay reward, depending on whether particle lengths or roughage sources were compared) was introduced as an explanatory variable. Individual cross points corresponded to the values of FR where the expected proportion *p* = 0.5 and differed across animals. Individual cross points were calculated as: *cp  = −α/β*, where *α* and *β* are an animal's estimated intercept and slope on the logit scale. The overall cross point was defined as the median of the cross points of all animals in the target population and estimated by the median of the individual cross point of the animals in the experiment. The overall cross point was compared to the midpoint (i.e. 21) using Wilcoxon's signed rank test, applied to the differences between the individual cross points and the midpoint, and an associated 0.95-confidence interval for the overall cross point was constructed.

**Figure 2:**
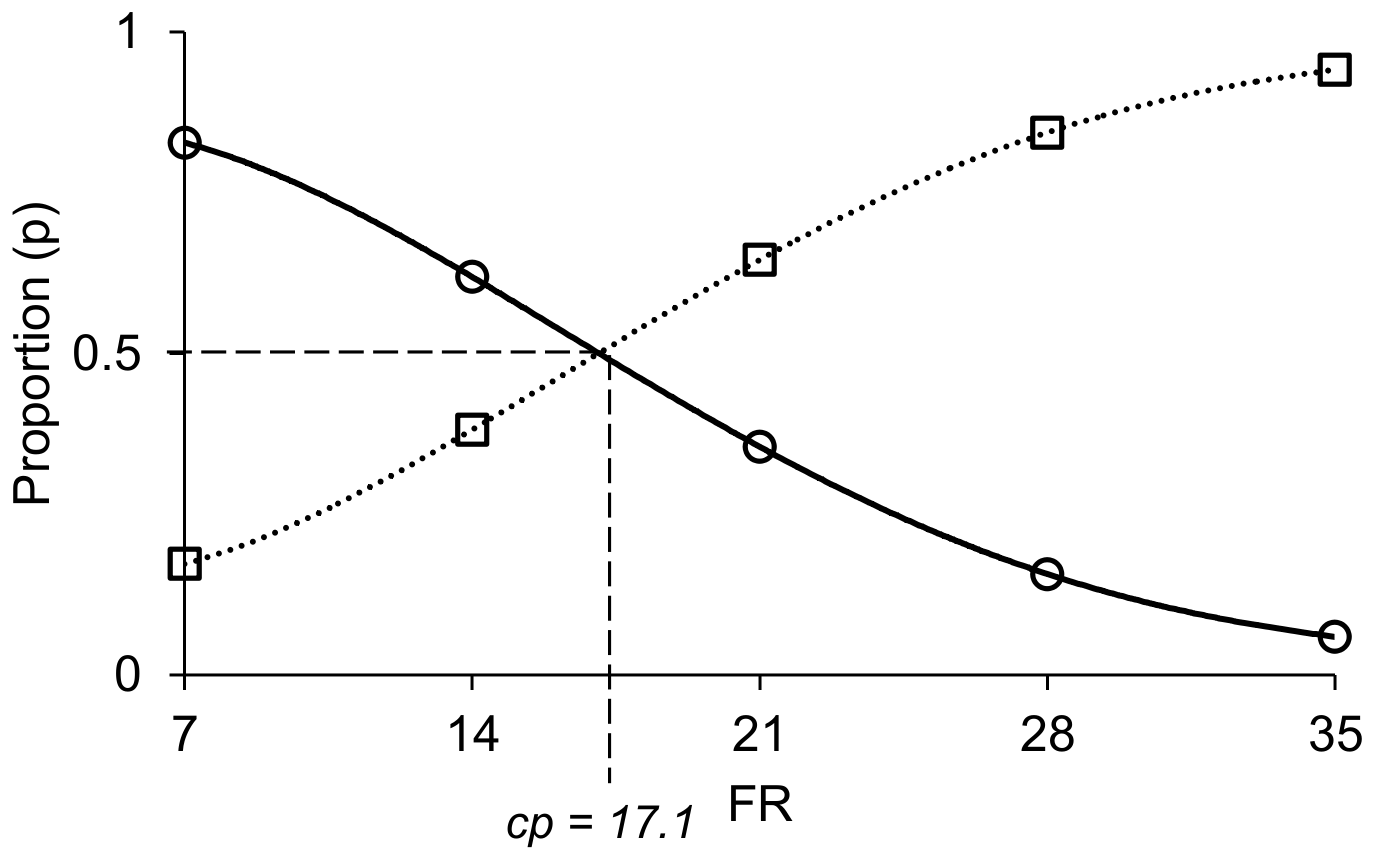
Cross point analysis illustrated. Graphical representation of the cross point (cp) of calf no. 2 for the comparison chopped hay (circles) versus long hay (squares) using proportions (p) of chopped hay rewards over total number of rewards. The proportions for long hay rewards were calculated as 1 - p. The x axis shows fixed ratio (FR) values for the chopped hay (the long hay fixed ratio values are 42 - FR). The lines connecting the points are 4^th^ order polynomials.

In order to demonstrate the meaning of “cross point” when using proportions instead of counts, a graphical representation, plotting predicted proportions of chopped hay rewards against FR for chopped hay, is shown for calf no. 2 (Figure 2). The curves fitted by proportions are sigmoid, and the curve for long hay is the opposite (*1-p*) of the curve for chopped hay (*p*). The cross point corresponds to the point where *p* = 0.5, which in this figure is illustrated by the intersection between the two curves (Figure 2).

P-values lower than 0.05 were considered significant. Calculations were conducted using SAS version 9.2 [32] and Genstat version 15 [33].

## Results

At the end of the study, calves weighed 248.4±5.9 kg on average, with an average daily gain of 1.5±0.1 kg/d. Roughage intake in the home pen during the weekend is shown in Table 1.

**Table 1:** Roughage intake in the home pen (mean ± SEM g/d).

Period	Age (wk)	Chopped hay	Long hay	Chopped straw	Long straw	Lucerne hay
Start^1^	7–9	106±22	216±12	83±12	93±9	366±41
Training^2^	9–15	362±49	355±55	266±32	142±17	
Testing^3^	15–21	505±55	423±56	238±84	316±30	

1 Roughage was provided ad libitum during the habituation period, one roughage type at a time.

2 Roughage was provided ad libitum, one roughage at a time (2 days per week without training).

3 Roughage was provided ad libitum, two roughage types at a time (1 day per week without testing). The two types of roughage provided were from the same source but had different particle lengths.

### Double demand and cross points

The nine calves used in the present study were successfully trained to work economically on two panels delivering the same roughage reward (i.e. Lucerne hay), in that they consistently chose the panel with the lowest workload more often than the other panel (Table 2). Moreover, all calves were motivated to work for both hay and straw rewards throughout the study, despite high milk replacer and concentrate provision in the home pen (Table 3).

**Table 2:** Cross points of individual calves for each comparison, including training.

Calf	Training	Chopped vs. long hay	Chopped vs. long straw	Hay vs. straw
1	18.5	14.2	6.7	30.8
2	25.1	17.1	22.2	33.8
3	22.7	18.9	22.5	27.5
4	23.2	13.8	21.6	42.3
5	18.6	12.2	19.9	33.5
6	20.8	17.9	20.8	38.9
7	25.9	14.5	6.8	41.4
8	17.0	14.3	30.9	117.4
9	21.7	19.3	20.7	46.1
Median	21.7	14.5	20.8	38.9
Confidence interval	18.9–23.9	14.0–18.0	13.8–25.4^1^	32.3–42.0^2^
P-value	0.734	0.004	0.910	0.004

1 Note that the confidence interval here includes 21 and is wide, indicating a large variation between individual calves and a difficulty in drawing conclusions on this particular comparison.

2 Note that 42.0 is the largest value that the upper bound can take, since larger values would correspond to negative values for 42-x for the other resource.

**Table 3:** Total median number of rewards achieved (and total grams).

Comparison	FR	Median	Q1^3^	Q3^3^
Chopped vs. long hay^1^	7–35	57.0 (285)	45.0	86.5
	14–28	27.5 (138)	22.0	45.0
	21–21	26.0 (130)	15.0	42.0
	28–14	49.0 (245)	25.0	58.0
	35–7	81.5 (408)	45.0	100.8
Chopped vs. long straw^1^	7–35	22.0 (110)	11.0	43.0
	14–28	19.5 (98)	12.0	25.0
	21–21	17.0 (85)	10.0	24.0
	28–14	15.5 (78)	10.0	24.0
	35–7	31.0 (155)	17.0	52.0
Hay vs. straw^2^	7–35	79.7 (638)	60.6	105.8
	14–28	46.0 (368)	31.7	78.0
	21–21	28.1 (225)	19.0	36.0
	28–14	24.0 (192)	16.4	33.8
	35–7	18.7 (150)	14.6	31.6

1 Reward size was 5 g.

2 Reward size was 8 g.

3 1^st^ and 3^rd^ quartile for the median.

Calves showed a preference for long hay over chopped hay, indicated by an overall median cross point below the midpoint 21 and different from the midpoint (Table 2). The overall cross point for the comparison chopped straw versus long straw was not different from the midpoint (Table 2), which indicates that calves showed no preference for chopped or long straw. However, the confidence interval was wide, indicating large variation between individuals, and three calves seemed to have expressed a preference for chopped straw (calves no. 1 and 7) or long straw (calf no. 8) (Table 2). Calves showed a preference for chopped hay over chopped straw, indicated by an overall cross point higher than the midpoint, and different from the midpoint (Table 2). The cross point, i.e. 38.9, is higher than 35, which is the highest FR that was imposed in the present study, indicating that calves always achieved more hay rewards than straw rewards regardless of the costs. Median number of rewards consumed during one session was highest for the comparisons including hay rewards, and higher when the preferred resource was available at a low price for the comparison chopped versus long hay, and hay versus straw, i.e. comparisons where one resource was preferred over the other (Table 2).

### Post-mortem results

The calves in the present study had no overt health problems during the experiment. The results of the post-mortem gastrointestinal and respiratory health measurements showed no severe pneumonia, no rumen hyperkeratinisation, and relatively good rumen development (rumen development score [median]  = 3.0). The median abomasal lesion score was 4.0 and was close to that found in European veal farms with large numbers of animals [34].

## Discussion

The main aim of this study was to investigate the preferences of calves for different roughage particle lengths. Relative preference was quantified using a double demand operant conditioning paradigm. Double demand operant conditioning has previously been applied to rats [24], [28], [30], chickens [35], pigs [23], [29], and adult cattle [36], but we could not find a study applying the double demand approach to calves. The methodology used to train the calves in the present study took 6 weeks in total, starting with 9 week-old calves (training started 2 weeks after the arrival of the calves, the first two weeks being used to familiarise calves to the roughages). The results showed that calves fed a high energy diet were willing to work for extra roughage rewards, including Lucerne hay, good quality hay and barley straw. The calves adjusted their efforts on the two panels according to their respective price such that when the two panels yielded the same roughage (Lucerne hay), they obtained more rewards from the panel with the lowest cost in all sessions. Calves expressed their preferences when two different rewards where available. It was possible to quantify the strength of preferences via the deviation of the cross point from the midpoint. This is clearly seen when comparing the deviations found for the preference of long hay over chopped hay (deviation of 6.5 from the midpoint) and the preference of hay over straw (deviation of 17.9 from the midpoint). This suggests that the preference of hay over straw is stronger than that of long hay over chopped hay in calves. Hay differs from straw in a number of ways apart from structure, as it contains more energy [22], has a different flavour [37] and is thought to have a beneficial influence on rumen function: due to increased fermentation, hay should lead to better papillae development [26]. However, the latter effect may be minimal in this study because of the high level of concentrate fed. The cross point for the comparison of hay versus straw was above 35, which is the highest cost imposed on resources in the present study. This indicates that for this comparison, the range of costs did not include a large enough difference in values. However, the results obtained do seem to confirm the hypothesis that hay is a preferred roughage compared to straw, even when energy is no limiting factor.

The statistical method used in this paper for cross point analysis of double demands differs from methods used in previous studies [23], [24], [28]–[30]. The presently applied method considers three aspects in the analysis of double demand functions. First, the dependence between data for the two resources offered simultaneously is included by using proportions as a response variable. Second, individual variation is expressed in an accessible and clear manner, and looking at individual cross points offers a clear picture of variation in preferences across animals [24], [28]. Third, the analysis is robust, that is, not critically dependent upon complex model assumptions, and the use of Wilcoxon's signed rank test offers a conceptually and computationally straightforward statistical method.

Calves did not consistently prefer the roughage associated to the shortest ingestion and digestion time, i.e. chopped roughage; they did show a preference for long hay over chopped hay, but no preference was apparent for either long straw or chopped straw. Calves in this study were fed a high energy diet, consisting of milk replacer and concentrate, between testing sessions. It was, therefore, expected that these calves would not necessarily show a preference for the roughage permitting the best rate of energy gain. Furthermore, calves did not “abandon” the panel with the highest workload. This was the case when both panels provided the same reward, as well as when the “cheap” panel delivered the preferred reward. Contrafreeloading describes the concept where animals work for food when the same food is simultaneously freely available [2], [38]–[40]. Although the food in the present study was never “free”, it was sometimes very “cheap”. Therefore, the animals displayed something very close to contrafreeloading, that we could term contracheaploading, and which most likely stems from the same motivations. Previous studies using double demand also observed this behaviour in their animals [29], [30]. Contracheaploading in double demand operant setups most likely signals information gathering from various available resources, just like contrafreeloading [38] and could be an indication of animals' adaptation to a changing environment, e.g. the depletion of the highest quality food patch [2], [38], [39]. In nature, food patches used by animals will deplete over time, and gathering information about alternative patches may increase survival over the long term. In the present set-up the relative cost of the two resources were alternated between daily sessions and thus there was a high level of uncertainty, which is hypothesised to increase contrafreeloading [2]. In other contexts, contrafreeloading could be an indication of animals' need to express appetitive behaviour [10]. However, since calves had to work for all roughage resources, this is an unlikely explanation in the present set-up.

The preference for long hay found in the present study could be explained in two non-mutually-exclusive manners. First, calves may have preferred long hay because it required more chewing, and calves may have a high motivation for performing this behaviour [10]. The calves may have perceived the long hay portion as being larger than the chopped hay portion, through increased eating time [19], increased rumen fill [17], and slower clearance rate of the reticulorumen [20]. Long hay may also increase rumination as a post-ingestive consequence [13], [41]. During the habituation period and in the home pen on days without training or testing, calves were fed each roughage type on separate occasions, which is assumed to have been sufficient for calves to learn post-ingestive consequences of all roughage types, including consequences for rumination [42].

Second, calves may have preferred long hay because it resulted in improved rumen function compared to the chopped hay, given that calves were indeed aware of post-ingestive consequences of each particle length. Longer particles of roughage take longer to chew and ruminate before the particle length is sufficiently reduced to move from the reticulorumen to the abomasum, and increased rumination increases salivation [7], [19]. Saliva secretion increases the buffering capacity of rumen fluid [7], [19], and prolonged presence of roughage particles in the rumen improves rumen motility and stimulates the removal of ingested hair and small feed particles from the rumen papillae [43]. This is especially important in calves fed large quantities of concentrate, and for which access to roughage is restricted. Therefore, longer roughage particles improve rumen muscularisation, papillae development, and rumen osmolality and pH [15], [16], while preventing hairball and plaque development [13], [21], [43].

Interestingly, calves showed a preference for long over chopped roughage for hay but not for straw. Given the large variation between calves found in the comparison of chopped versus long straw (illustrated by the 95% confidence interval), it is difficult to conclude on this particular result. It is possible that with a larger sample of animals, a preference for one of the straws would have been observed. Straw is a coarse and low quality roughage with low energy and high fibre content, resulting in a low rate of energy gain [22]. Preference for shorter particles of straw was found to be stronger compared to preference for shorter particles of high quality roughage (such as hay) in sheep [22]. Therefore, ruminants can show preferences for different structures, even with low quality roughages. In our study, given the high energy feeding strategy provided outside of testing, calves were expected to show a preference for longer particles. Since this preference was not found for straw, we can only speculate that long straw was associated with some sort of cost that outweighed the benefits, and that this cost was not present, or present to a lesser extent in long hay. A possible cost could be worse abomasal damage [44]. Abomasal damage, i.e. lesions on the abomasal wall, could result from a combination of three factors: a) overfilling of the abomasum because of large milk meals causing local loss of blood supply of the abomasal wall (ischaemia), b) exacerbation of this damage from poorly digested feed particles coming from a poorly developed rumen, and c) exacerbation of this damage by coarse feed stuffs [13], [45], [46].

The post-mortem health measurements were carried out in the present study to check whether calves were healthy, and whether any underlying health problems could have explained any of the preferences. The feeding strategy combined with possibility to work for roughage in the operant pen aimed to permit a good growth, and this was successfully achieved. Looking at the numbers, rumen development seemed better than that found in European veal calves, but abomasal damage appeared comparable [34]. Similar abomasal damage could indicate that milk feeding was an important factor in causing abomasal damage [45], or that the improvement in rumen development was insufficient to minimise abomasal damage in the current study [46]. The infrequent feeding of large amounts of milk replacer in the present study may have caused the observed abomasal damage [45] (and could have further caused other physiological problems, such as for example insulin resistance [47], [48], although this is not thought to have affected the results in any way). It is not known how abomasal damage may affect the preference for long or chopped particles of roughage. Despite these potential health issues, this feeding strategy was chosen to enable good control of milk intake (in terms of amount and time) before testing, in order to reduce inter- and intra-calf variation.

## Conclusions

The present findings showed that 2–5 month old calves can learn a double demand operant setup and are motivated to work for roughage in addition to a high energy diet comprising of milk replacer and concentrate. Overall, calves preferred long particles of hay, but not straw, compared to chopped, and calves had a strong preference for chopped hay over chopped straw. These findings support the idea that ruminants are able to make choices based on rumen function and possibly also based on their motivation to chew and ruminate. These findings could be used to improve the welfare of calves in production systems: Farmed calves fed high energy diets alongside hay might benefit (e.g. in terms of rumen function) from being offered long hay instead of chopped hay.
